# Spatiotemporal Transmission Patterns and Determinants of Dengue Fever: A Case Study of Guangzhou, China

**DOI:** 10.3390/ijerph16142486

**Published:** 2019-07-12

**Authors:** Yebin Chen, Zhigang Zhao, Zhichao Li, Weihong Li, Zhipeng Li, Renzhong Guo, Zhilu Yuan

**Affiliations:** 1 School of Resource and Environmental Sciences, Wuhan University, Wuhan 430072, China; 2 Research Institute of Smart City, School of Architecture and Urban Planning, Shenzhen University, Shenzhen 518060, China; 3 Ministry of Education Key Laboratory for Earth System Modeling, Department of Earth System Science, Tsinghua University, Beijing 100084, China; 4 School of Geography, South China Normal University, Guangzhou 510631, China; 5 Institute of Digital Agriculture Research, Fujian Academy of Agricultural Sciences, Fuzhou 350003, China

**Keywords:** dengue fever, spatial interactions, geo-detector, determinants

## Abstract

Dengue fever is one of the most common vector-borne diseases in the world and is mainly affected by the interaction of meteorological, human and land-use factors. This study aims to identify the impact of meteorological, human and land-use factors on dengue fever cases, involving the interplay between multiple factors. The analyses identified the statistically significant determinants affecting the transmission of dengue fever, employing cross-correlation analysis and the geo-detector model. This study was conducted in Guangzhou, China, using the data of confirmed cases of dengue fever, daily meteorological records, population density distribution and land-use distribution. The findings highlighted that the dengue fever hotspots were mainly distributed in the old city center of Guangzhou and were significantly shaped by meteorological, land-use and human factors. Meteorological factors including minimum temperature, maximum temperature, atmospheric pressure and relative humidity were correlated with the transmission of dengue fever. Minimum temperature, maximum temperature and relative humidity presented a statistically significant positive correlation with dengue fever cases, while atmospheric pressure presented statistically significant negative correlation. Minimum temperature, maximum temperature, atmospheric pressure and humidity have lag effects on the transmission of dengue fever. The population, community age, subway network density, road network density and ponds presented a statistically significant positive correlation with the number of dengue fever cases, and the interaction among land-use and human factors could enhance dengue fever transmission. The ponds were the most important interaction factors, which might strengthen the influence of other factors on dengue fever transmission. Our findings have implications for pre-emptive dengue fever control.

## 1. Introduction

Dengue fever (DF) is an acute mosquito-borne disease caused by dengue virus and transmitted by *Aedes* mosquitoes [1]. This disease has a high incidence rate, rapid transmission speed and high mortality rate in critically ill patients [2]. The clinical manifestations of DF patients are fever, severe headache, muscle and joint pain and some patients have lymphadenopathy, rash and even bleeding tendency [1,3,4]. The global spread of DF has caused serious economic losses to people living in tropical and subtropical countries [1,5]. Evidence has shown that more than 100 countries and regions comprising 2.5 billion people are facing the threat of DF, which poses great challenges to global public health [6,7,8,9]. 

In mainland China, DF cases have been reported every year since 1997, especially in Guangdong Province. The DF incidence rate in 2014 was the highest in the Guangdong Province in the past 30 years, and Guangzhou city (the capital and most populous city of Guangdong Province) is the most seriously affected area. In 2014, 37,386 confirmed DF cases were found in Guangzhou city, accounting for 82.67% of the total number of DF cases in Guangdong Province. *Aedes albopictus* and *Aedes aegypti* are the main DF vectors in Guangzhou [10]. Referring to mosquito breeding and DF transmission, meteorological conditions [11,12] as well as human (demographic structure, population size) [13,14,15] and land-use factors (traffic, standing water) [16,17] have been identified as important factors affecting the transmission of DF disease. For example, mosquito breeding activities are affected by temperature: *Aedes albopictus* are most active in breeding activities when the temperature is between 26 °C and 30 °C, but the feeding activity of mosquitoes decreases and mortality increases when the temperature is lower than 15 °C or higher than 35 °C [12]. Rainfall and humidity are positively associated with the reproduction of *Aedes* at the initial life stage of *Aedes* mosquitoes [18]. Poor sanitation, wet areas and densely populated areas are ideal places for *Aedes* mosquito breeding [1,19]. 

Patterns and determinants relating to DF transmission have been investigated in many studies. However, existing studies neglect the interactions between impacting factors when examining the statistical relationships between factors (e.g., meteorology, human factors, etc.) and DF transmission [6,13]. Moreover, when constructing models to identify the spatial patterns of transmission and its determinants, most studies only investigate the effect of each factor on DF at the local level (e.g., using a geographically weighted regression model), without examining the interactions between multiple factors from a spatial perspective as well as incorporating the interplay of multiple factors in spatial models [20]. The spatiotemporal transmission of DF is the product of a multifactorial combination. The spatial interaction among various factors may strengthen the process of DF transmission [1]. Therefore, it is necessary to examine the spatial interactions between factors before examining the effect of factors on DF cases. 

Within this context, this study aims to identify the impact of meteorological, human and land-use factors on DF cases, incorporating the spatial interactions between multiple factors. We employed the cross-correlation model to examine the impact of meteorological factors on DF transmission and the geo-detector model to examine the spatial interactions between human and land-use factors, and then investigated the impact of these factors and their interactions on DF transmission. Moreover, we conducted DF spatiotemporal transmission characteristics analysis in Guangzhou to clarify the potential impact of various factors on DF in detail, which lays the foundation for further identifying the impacting factors. The findings of this study could provide an important reference for prevention and control in cities with a high risk of DF.

## 2. Materials and Methods 

### 2.1. Study Area

**Figure 1:**
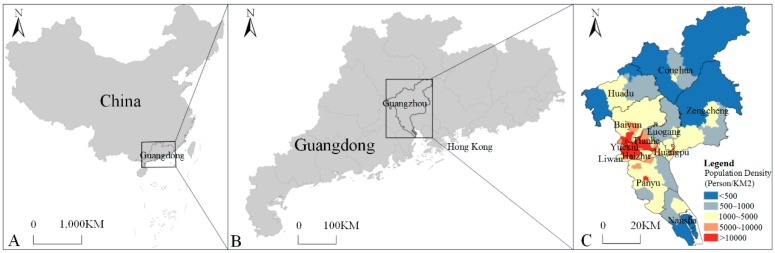
Extent of the study area. (**A**) The location of Guangdong Province in China; (**B**) the location of the study area in Guangdong Province; (**C**) the extent of the study area and population density distribution.

Located in the south-central part of Guangdong Province in China, Guangzhou (112°57′–114°3′ E, 22°26′–23°56′ N) is composed of Yuexiu, Liwan, Haizhu, Tianhe, Baiyun, Luogang, Huangpu, Panyu, Huadu, Nansha, Zengcheng and Conghua districts and had a resident population of 14.49 million and an overall area of 7434.4 km^2^ in 2014 (Figure 1). The average annual temperature in Guangzhou in 2014 was between 18.7 and 26.7 °C; the hottest month was July, and the coldest month was January. The rainy season was concentrated in April to June, and the annual average relative humidity was 81%. However, due to the obsolete urban drainage system in the old city center (Yuexiu district), the annual flood season from April to June was prone to produce urban waterlogging, which provided conditions for mosquito breeding.

Guangzhou was selected as the study area because it has always been the hardest hit area of DF in Guangdong Province and China [10]. Since 2001, the DF epidemic in Guangzhou has gradually intensified. Large-scale DF outbreaks happened in 2002, 2006 and 2014, and the outbreak in 2014 was the most serious one. The cumulative reported cases in 2014 reached 37,386 cases, which was the most serious DF epidemic in Guangzhou in the past 30 years. Guangzhou is the center of transportation, finance, industry and trade in southern China and has frequent economic and cultural communication with the nations of Southeast Asia and Africa, which also increased the importation risk of DF. 

### 2.2. Data Collection

#### 2.2.1. Dengue Fever Data

DF cases were confirmed by epidemiological investigation and laboratory diagnosis. Patients suspected of having DF included those who lived or traveled to DF endemic areas (over 14 days) and subsequently exhibited clinical symptoms such as fever, with two or more of these manifestations: nausea, vomiting and skin erythema. Medical professionals were responsible for the clinical classification of the disease, along with laboratory confirmation through serology, NS1 (non-structural protein 1) test or polymerase chain reaction (PCR) tests [21]. Acute phase sera were tested by enzyme linked immunoassay (ELISA) for the detection of dengue NS1 antigen and dengue IgM antibodies. Convalescent phase sera were also tested by dengue IgM ELISA to identify seroconversions. Acute phase sera from suspected patients who were positive by NS1 ELISA or IgM ELISA in either the acute or the convalescent phase sera were also tested by reverse transcriptase polymerase chain reaction (RT-PCR) to identify the infecting serotype [22]. We defined a confirmed case of DF as a DF-suspected patient with a positive NS1 ELISA, acute or convalescent phase IgM ELISA or RT-PCR. The Chinese Ministry of health lists DF as a compulsory notification disease. According to the guidelines for DF case surveillance, county-level disease prevention and control agencies should record all DF cases and send them to the Information System for Notifiable Disease (ISND).

**Figure 2:**
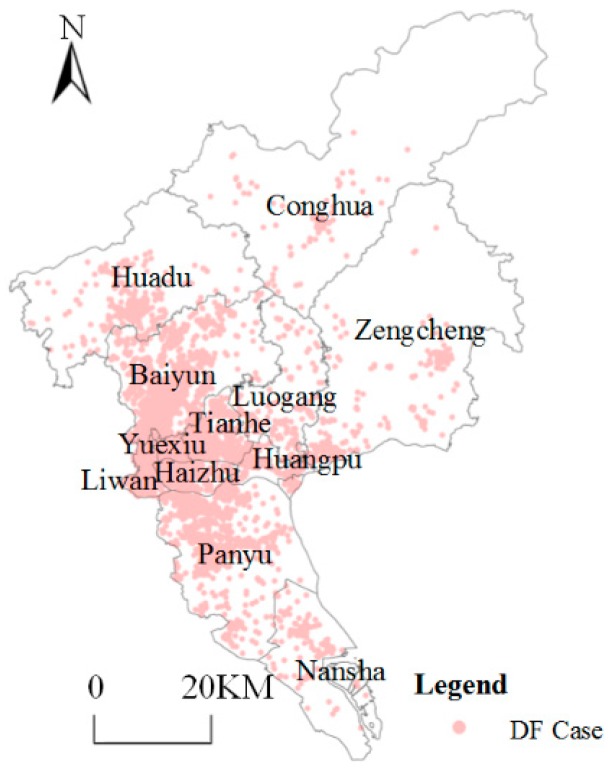
Spatial distribution of dengue fever (DF) cases.

In this paper, the data of confirmed cases of DF were provided by Guangdong Provincial Center for Disease Control and Prevention, including all the DF confirmed cases of Guangzhou reported in ISND, totaling 37,386 cases from 1 January 2014 to 31 December 2014. Data structures of DF cases included the onset date, residence address, longitude and latitude attributes. Data were processed using ArcMap 10.2 (Environment System Research Institute, Inc., New York, NY, USA) to obtain the DF spatial distribution (Figure 2).

#### 2.2.2. Factor Data

Table 1 shows the data used in this study, including the meteorological data, human data and land-use data.

**Figure 3:**
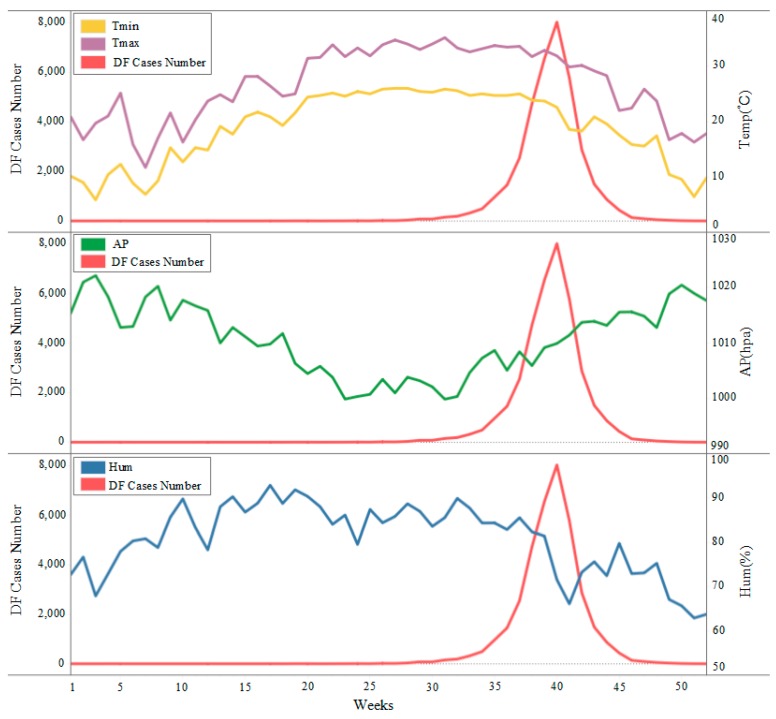
Sequence of weekly DF cases and meteorological factors in Guangzhou, 2014.

The meteorological data were provided by the Wushan meteorological monitoring station in Guangzhou, including daily minimum temperature (Tmin), maximum temperature (Tmax), atmospheric pressure (AP) and relative humidity (Hum) data, with a total of 365 records. Daily meteorological data were used to calculate the weekly average value (Figure 3), and the data were presented in Microsoft Excel 2013.

The land-use data were extracted from a 2.5 m SPOT (Système Probatoire d’Observation de la Terre) satellite remote sensing image and Baidu map (https://map.baidu.com) in 2014 by combining an automatic extraction algorithm (i.e., the Modified Normalized Difference Water Index, MNDWI) with manual vectorization, including roads, subways, ponds and residential areas, and the results of extracted land-use data were superimposed with images for verification. Roads include highways, national highways, provincial highways, urban expressways and county roads. Ponds refer to water areas with different sizes (e.g., <1000 m^2^) and a relatively regular shape. Residential areas refer to residential buildings.

The human data include population and community age data. The data of population size were collected from the Guangzhou statistics bureau, and the points of interest (POI) of the residential community were collected from the Baidu map (https://map.baidu.com), which is the most popular map engine in China. The residential community POI included the location and the building time attributes, and the community age refers to the length of time from the time the community was built to 2014. The older the community, the worse the infrastructure and sanitation, which was more likely to lead to DF transmission [1]. The population data were distributed to each building according to the ratio of the total area of buildings (accumulated by the plane area of each floor) and the building area, while avoiding the homogenization problem that was caused by traditional sampling methods and improving the accuracy of the population data. 

### 2.3. Methodology

The paper aimed to analyze the impacts of meteorological factors, human factors and land-use factors on DF transmission. The analysis was divided into two parts. Firstly, we adopted cross-correlation analysis to analyze the impacts of meteorological factors (including minimum temperature, maximum temperature, relative humidity and atmospheric pressure) on the DF transmission given that meteorological factors have temporal characteristics. Secondly, the geographical detector model (geo-detector) was employed to analyze the interaction between human factors and land-use factors on DF transmission. In addition, we conducted DF spatiotemporal transmission characteristics analysis in Guangzhou to clarify the DF hotspots and help identify the potential impact of various factors in detail.

#### 2.3.1. Cross-Correlation Analysis

DF transmission mainly depends on mosquito breeding activities. Meteorological factors, such as temperature, relative humidity and atmospheric pressure, could affect the breeding activities of mosquito vectors [1,12,18,23]. Increased temperature could stimulate the breeding activity of mosquito vectors; increased rainfall and relative humidity could increase mosquito breeding sites, and decreased atmospheric pressure could increase the contact probability of *Aedes* mosquitoes and people. Therefore, we selected minimum temperature, maximum temperature, relative humidity and atmospheric pressure to study the impact of meteorological factors on DF transmission.

Cross-correlation analysis can be used to analyze the correlation between meteorological factors and DF with different time lags [24]. In order to study the impact of temperature, relative humidity and atmospheric pressure on DF transmission, the cross-correlation analysis method was adopted to study the impact of the minimum temperature, maximum temperature, relative humidity and atmospheric pressure on DF transmission with different weekly lags (with the maximum delay time set as 12 weeks [25]). The related operations were completed in SPSS 22 V22.0 (IBM, Armonk, NY, USA).

#### 2.3.2. Geo-Detector Analysis

Human and land-use factors were also key factors affecting mosquito breeding, including population size, traffic network density, the distribution of aging infrastructure and standing water. The increase of population size and traffic network density could increase the risk of healthy people being exposed to dengue carriers and *Aedes* mosquitoes [16], and the distribution of old city infrastructure and standing water affected the spatial distribution of the *Aedes* mosquitoes [1,23,24]. Therefore, this study selected population size, road network density, subway network density, community age and pond distribution to study the impact of human factors and land-use factors on DF.

The grid method and geo-detector analysis were conducted to explore the relationship between DF and various potential factors from a microscopic perspective using the data of DF and the potential driving factors. The study area was divided into 154 rows × 112 columns by using a 1 km × 1 km grid (according to the flight distance of mosquitoes; it is generally believed that the range of the mosquito’s activity is mainly within 100 m of its birthplace, and the maximum is no more than 1 km [23]), making a total of 7745 grids. The DF cases and the 5 potential driving factors—population, community age, subway, roads and ponds—were spatialized into each grid using ArcGIS 10.2 (Environment System Research Institute, Inc., New York, NY, USA). 

A factor-detector is a submodule of the geo-detector [26,27,28] which was used to identify the effects of each factor on the transmission process of DF. The factor-detector model can be written as follows:

(1) $$q_{D,H}=1-\frac{1}{n\sigma_{H}^{2}}\sum_{i=1}^{m}n_{D,i}\sigma_{H_{D,i}}^{2}$$

where $q_{D,H}$ is the influence value of factor D on DF H; $\sigma_{H}^{2}$ is the variance of DF throughout the area; n is the number of samples in the study area; m is the number of secondary areas; $\sigma_{H_{D,i}}^{2}$ is the variance of the DF number in the secondary level, when $\sigma_{H_{D,i}}^{2}$ is not equal to 0, and the model is constructed. $q_{D,H}\in \left[0,1\right]$, when $q_{D,H}=0$, it indicates that the number of DF is not affected by factors. The larger the $q_{D,H}$, the greater the ability to explain the transmission process of DF. When $q_{D,H}=1$, the interpretation effect is optimal. The interaction detector is also a submodule of the geo-detector which can explore the interaction effect among factors. The model can be written as follows:

(2) $$q_{D,H}\left(D_{i}\cap D_{j}\right)>q_{D,H}\left(D_{i}\right)+q_{D,H}\left(D_{j}\right)\;:\;\mathrm{Nonlinear}\;\mathrm{synergy}$$

(3) $$q_{D,H}\left(D_{i}\cap D_{j}\right)<q_{D,H}\left(D_{i}\right)+q_{D,H}\left(D_{j}\right):\;\mathrm{Nonlinear}\;\mathrm{antagonism}$$

(4) $$q_{D,H}\left(D_{i}\cap D_{j}\right)=q_{D,H}\left(D_{i}\right)+q_{D,H}\left(D_{j}\right):\;\mathrm{Independence}$$

## 3. Analyses and Results

### 3.1. Spatiotemporal Transmission Characteristics Analysis

Figure 3 describes the time distribution characteristic of DF. From January to May (1–21 weeks), DF cases appeared slowly, and only 8 cases occurred during this period. From June to October (22–43 weeks), the DF cases number increased rapidly, with a total of 36,170 cases. DF showed an outbreak trend in this stage. From November to December (44–52 weeks), the number of DF cases decreased.

**Figure 4:**
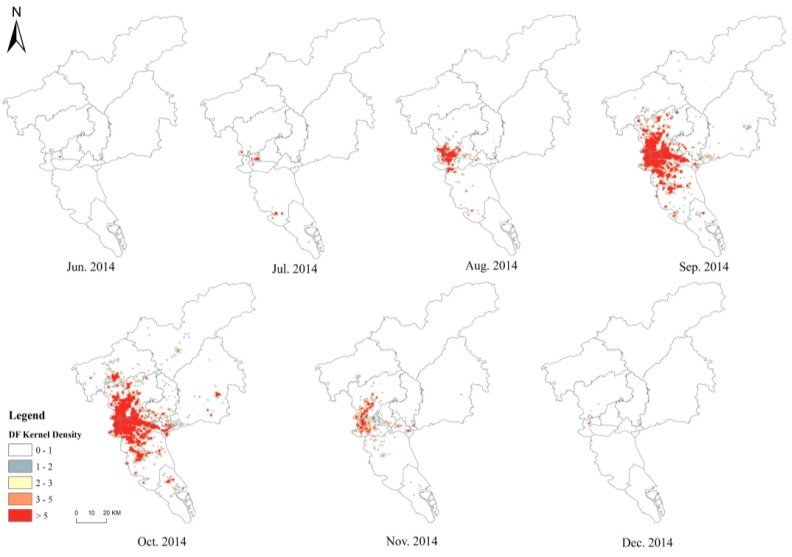
Transmission characteristics of DF cases.

Figure 4 shows the monthly spatiotemporal hotspot distribution of DF. It highlights that the DF initially tended to spread into the old city center (Yuexiu district), thus exhibiting obvious aggregation characteristics, and then spread rapidly to surrounding areas—Liwan, Haizhu, Baiyun and Tianhe districts—showing the characteristics of “high center and low periphery”, and finally covering the entire city. 

**Figure 5:**
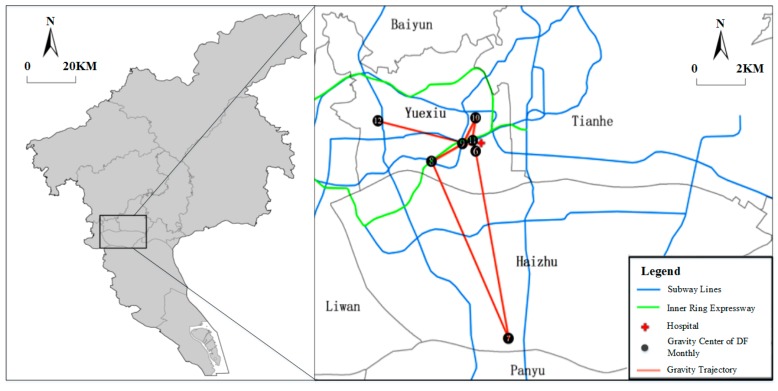
Distribution of the monthly gravity center of DF.

Figure 5 describes the monthly gravity center of DF, which indicates that monthly gravity centers of DF were mainly concentrated in the southeastern part of Yuexiu District.

### 3.2. Relationship between Dengue Fever and Driving Factors

#### 3.2.1. Meteorological Factors

Table 2 shows the cross-correlation results between DF cases and meteorological factors. It highlights that the weekly average minimum and maximum temperature had significant positive effects on the number of DF cases with a lag of 0–12 weeks. The minimum temperature with a 9-week lag had the strongest impact on DF transmission with a correlation coefficient of 0.945 and the maximum temperature with a 7-week lag had the strongest impact with a correlation coefficient of 0.917. The weekly average atmospheric pressure had a significant inverse effect on the number of DF cases with a lag of 0 to 12 weeks, and the effect of atmospheric pressure on the DF transmission was strongest at the lag of 11 weeks with a correlation coefficient of −0.92. The weekly average relative humidity had a positive effect on the DF transmission with a lag of 8–12 weeks. 

#### 3.2.2. Human and land-Use Factors

Table 3 shows the geo-detector results. We find that 5 factors, including population, community age, subway, road and pond, have significant impacts on the DF distribution. The population (0.624), community age (0.382) and subway (0.134) have an important impact on the DF distribution, followed by roads (0.05) and ponds (0.001).

As shown in Table 4, the interaction among land-use and human factors strengthens DF transmission, while ponds, the subway and roads were the important interaction factors. Ponds ∩ roads = 0.058 (↑9.4%) (∩ means the interaction-effect between factors; ↑ means the increased ratio of factor interaction-effects to the simple increase of factors); ponds ∩ subway = 0.146 (↑6.6%); and ponds ∩ community = 0.388 (↑0.78%).

## 4. Discussion

The analysis of the spatiotemporal transmission characteristics of DF indicated that June to October was the main period of DF transmission, and the DF hotspots were mainly concentrated in the southeast of Yuexiu district. In 2014, April to June was the rainy season in Guangzhou and the accumulated rain days reached 54 days during the period. Due to the aging infrastructure and poor drainage system, a large amount of water accumulated in Guangzhou. In addition, the average temperature of Guangzhou from April to June remained between 22 °C and 28 °C, which was conducive to the breeding of mosquito vectors and provided good conditions for DF outbreak (June to October). A further investigation of the DF hotspots of Guangzhou revealed the following characteristics. First, the hotspots had a convenient transportation network, which was close to the subway lines and the Guangzhou inner ring expressway in space, and there were several large hospitals around it, such as the First Affiliated Hospital of Guangdong Pharmaceutical College (Figure 5), which made residents living in the aggregation area more vulnerable to DF. Second, the buildings and infrastructure in the hotspot had gradually aged due to their long service life, which provided a good environment for mosquito breeding. In the aggregation area, most of the houses were old-fashioned buildings that had been built more than 30 years ago. The aggregation area had a high-building density, and the houses were occluded from each other and surrounded by a large number of trees and shrubs, thereby increasing the number of dark and humid places. Third, there were a large number of frail elderly and immigrant people from countries with high DF incidence, which increased the risk of DF transmission. 

The findings of the cross-correlation analysis showed that minimum temperature, maximum temperature, atmospheric pressure and relative humidity had a lagging effect on the DF transmission. The minimum temperature and maximum temperature had a positive lag correlation with DF transmission for 0–12 weeks, with the strongest correlation at 9 weeks and 7 weeks, respectively. Atmospheric pressure had a negative correlation with the DF transmission, and the maximum correlation value was at 11 weeks. Relative humidity had a positive correlation with the DF transmission for 8–12 weeks. This was because DF is an acute mosquito-borne disease transmitted in a “human–mosquito–human” mode [5], and there was a time-lag effect from the increase of mosquito population to the increase of DF cases. Therefore, the influence of temperature, atmospheric pressure and relative humidity on the DF transmission showed a corresponding time-lag relationship. DF is an acute mosquito-borne disease transmitted by *Aedes* mosquitoes, and the breeding of *Aedes* mosquitoes required appropriate temperature, relative humidity and atmospheric pressure conditions. In general, increased ambient temperature and relative humidity accelerated the reproduction of mosquito vectors and promoted the spread of DF [1,23]. The numbers of *Aedes larvae* were higher in the rainy season than in the winter seasons, which made it easy to develop DF in areas with high relative humidity. The same findings were indicated by Wongkoon [29]. Morin [2] and Xiang [30] stated that a maximum temperature ranging from 21.6 to 32.9 and a minimum temperature from 11.2 to 23.7 are optimal for DF transmission, which was coincident with the annual temperature range in Guangzhou. Atmospheric pressure had an adverse effect on the DF transmission because the lower atmospheric pressure promoted the mosquito vectors flying at lower altitudes, which increased the possibility of human exposure to mosquitoes and increased the transmission risk of DF, which was consistent with the research results of Bulto [31].

The findings of the geo-detector analysis revealed that population, community age and the subway were the important driving factors affecting the DF transmission, followed by roads and ponds. In 2014, the DF hotspots in Guangzhou were mainly distributed in the places with dense population, old infrastructure and developed transportation networks. The interaction among people, DF carriers and mosquito vectors in these areas was greatly enhanced and increased the DF transmission probability. In addition, with regard to older communities, the poor sanitation conditions, a high proportion of susceptible populations (e.g., the elderly) and residents who neglected the issues of DF prevention and control strengthened the risk of DF transmission. This result was in accordance with the findings indicated by Morin and Bakhsh [2,32]. The proportion of the elderly population living in the old community was relatively larger, which increases the susceptible population and strengthens the risk of DF transmission [29]. The subway and its catchment areas were crowded places, which increased the chances of human contact with DF carriers, greatly enhancing the risk of human infection with DF (similar findings were found by Sanna and Hsieh [16]). Li [8] referred to the spread of the epidemic being mainly along the high-density road network area, and a high-density road network is an important factor contributing to the direction and scale of DF epidemics. Houses located along roads produced conducive conditions for *Aedes albopictus* breeding sites, resting sites, blood feeding sites, oviposition sites and areas to disperse. Additionally, compared with rivers and other flowing water, ponds had the characteristics of low-fluidity or non-fluidity and were prone to water accumulation. In the absence of natural enemies of mosquito larvae such as fish, ponds were suitable for mosquito breeding.

It is worth noting that the impact of these factors was exerted on DF risk through multiple mechanisms, and the spatial interaction of human (e.g., population, community age) and land-use factors (e.g., subway, road and ponds) could enhance the spatiotemporal transmission of DF. Furthermore, ponds were an important interaction factor because they could strengthen the influence of other factors on DF transmission. Located in the subtropical coast, the rainy season in Guangzhou is hot, humid and rainy [10,17]. Due to the aging of the drainage system and other infrastructure, Guangzhou often encounters urban waterlogging after heavy rains, and a large amount of accumulated standing water often appear in and around ponds, which provides the necessary water environment for the breeding of *Aedes* mosquitos. When the areas with high population density and high susceptibility interact spatially with water-prone areas, it could increase the exposure of humans to *Aedes* mosquitoes and increase the risk of DF transmission. This might be the reason that the spatial superposition of driving factors will enhance the “human–mosquito–human” [5] transmission mode of DF and accelerate the transmission speed.

Of course, this study was not without limitations. First, we used the reported confirmed cases of DF to analyze the relationship between the spatiotemporal transmission patterns of DF and driving factors, which have implications for pre-emptive DF control. However, it should be noted that some DF cases (such as undetected DF cases) might not be included in ISND, particularly in areas with underdeveloped medical conditions [1,33]. Second, DF transmission may be affected by the age structure of urban residents [22]. However, due to the poor availability of data, we only replaced the population age structure factor with that of the community age. Considering these limitations were mainly caused by data availability, further work can focus on data collection and enrich the driving factors in this study. Moreover, landscape characteristics (e.g., compositional and configurational features) can affect the transmission of mosquito-borne diseases by affecting human density and movement, mosquito abundance and distribution and human-mosquito encounters, and landscape characterization via landscape metrics has been applied in the studies of mosquito-borne diseases, such as malaria in the Amazon [34]. This method might be used in dengue research for a deep understanding of the impact of urban landscape feature on dengue’s spatiotemporal transmission. Such information might be useful for improving the actual prevention strategies of dengue transmission.

## 5. Conclusions

This study has two major strengths. Firstly, we quantified the effects of meteorological, human and land-use factors on DF transmission and built a hierarchical factor system of DF. Meteorological factors including minimum temperature, maximum temperature and humidity have a positive effect on DF transmission, while atmospheric pressure has a negative effect. Furthermore, minimum temperature, maximum temperature, atmospheric pressure and humidity have a lag effect on DF transmission. The population, community age and subway were the most important driving factors affecting DF transmission, followed by roads and ponds. Secondly, we identified that the interaction among land-use and human factors could enhance DF transmission, and the pond factor was the most important interaction factor, which could strengthen the influence of other factors on DF transmission. 

## Figures and Tables

**Table 1:** The factor data used in the study.

Factor Type	Variables	Description	Unit
Meteorological Data	Minimum Temperature	Daily minimum temperature	°C
	Maximum Temperature	Daily maximum temperature	°C
	Atmospheric Pressure	Daily atmospheric pressure	hPa
	Relative Humidity	Daily relative humidity	%
Human Data	Population	Number of people on the building	-
	Community age	Time span from the completion of the residential community to 2014	Years
Land-use Data	Road	Road network density	km/km^2^
	Subway	Subway lines network density	km/km^2^
	Ponds	Ponds area	m^2^

**Table 2:** Cross-correlation coefficients between weekly DF cases and meteorological variables.

Lag Weeks	0	1	2	3	4	5	6	7	8	9	10	11	12
Tmin (°C)	0.480 **	0.551 **	0.624 **	0.703 **	0.765 **	0.801 **	0.849 **	0.894 **	0.913 **	0.927 **	0.945 **	0.935 **	0.890 **
Tmax (°C)	0.588 **	0.642 **	0.712 **	0.783 **	0.833 **	0.850 **	0.878 **	0.917 **	0.901 **	0.882 **	0.862 **	0.840 **	0.786 **
AP (hpa)	−0.360 **	−0.414 **	−0.488 **	−0.593 **	−0.649 **	−0.705 **	−0.787 **	−0.836 **	−0.859 **	−0.895 **	−0.916 **	−0.920 **	−0.890 **
Hum (%)	−0.203	−0.155 *	−0.07	0.017	0.049	0.139	0.239	0.273	0.329 *	0.42 **	0.495 **	0.493 **	0.523 **

Note: ** Correlation is significant at the 0.01 level (2-tailed); * Correlation is significant at the 0.05 level (2-tailed).

**Table 3:** Result of factor-detector analysis.

Factor	Population	Community Age	Subway	Road	Ponds
q	0.624	0.382	0.134	0.050	0.001
p	0.01	0.01	0.01	0.01	0.04

**Table 4:** Result of interaction detector.

Factor	Population	Community Age	Subway	Road	Ponds
Population	0.624				
Community Age	0.658	0.382			
Subway	0.643	0.421	0.134		
Road	0.640	0.413	0.183	0.050	
Ponds	0.625	0.388	0.146	0.058	0.003
